# Computed Tomographic Analysis of Associations Between Pulp Stones and Demographic and Tooth-Related Factors in a North China Population

**DOI:** 10.3290/j.ohpd.c_2667

**Published:** 2026-05-06

**Authors:** Qian Gao, Gege Li

**Affiliations:** a Qian Gao Physician, Department of Stomatology, Shandong Provincial Third Hospital, Shandong University, Jinan, Shandong, China. Designed the study, performed data acquisition, analysed the data, wrote the paper, and approved the final version of the manuscript.; b Gege Li Attending Physician, Department of Stomatology, Shandong Provincial Third Hospital, Shandong University, Jinan, Shandong, China.Performed the research, analysed the data, reviewed and edited the manuscript.

**Keywords:** associated factors, cone-beam computed tomography, North China population, pulp stones

## Abstract

**Purpose:**

To investigate the associations between pulp stone occurrence and age, gender, dental arch, and tooth types in the North China population using cone-beam computed tomography (CBCT).

**Methods and Materials:**

CBCT images were selected based on inclusion criteria: complete permanent dentition (28 intact teeth) and absence of systemic diseases. A total of 335 patients aged 20–40 years were included. Prevalence of pulp stones was compared across age, gender, dental arch, and tooth type. Associations with demographic and tooth-related variables were examined using generalised estimating equations (GEE).

**Results:**

Pulp stones were detected in 28.4% of patients and 6.2% of teeth. 92.6% of affected patients had multiple stones, and 53.4% exhibited complete bilateral symmetry. Prevalence was significantly higher in females than in males, and in the maxillary arch than in the mandibular arch (both *P* < 0.05). Molars, particularly maxillary first molars, were most frequently involved (*P* < 0.008). No significant left–right or age- related differences were observed (*P* > 0.05). GEE analysis indicated that the occurrence of pulp stones was 1.25 times higher in the maxillary arch than in the mandibular arch, 2.05 times higher in females than in males, 19.28 times higher in molars than in incisors, and 4.14 times higher in canines than incisors.

**Conclusion:**

Pulp stones were more frequently observed in females, maxillary teeth, and first molars, showing no significant association with age or arch side but a tendency for multiple and symmetric occurrence within individuals.

Pulp stones are calcified nodules that may occur in the coronal or root portion of the pulp, where they may be free, adherent or embedded into the dentin.^7,10^ Their size varies considerably, ranging from particles as small as 50 μm in diameter to large masses that may nearly obliterate the pulp cavity.^21,23^ In most cases, pulp stones are asymptomatic and require no specific treatment.^7^ Many epidemiological studies have used radiological methods to detect pulp stones.^5,8,12,25,36^

Cone-beam computed tomography (CBCT) delivers high-resolution images across multiple spatial planes, which confers superior accuracy in pulp stone identification and allows for the comprehensive assessment of the location and extent of calcification,^30^ thereby circumventing the inherent limitations of two-dimensional imaging modalities.^29^

The aetiology of pulp stone formation remains not fully understood. Local oral factors and systemic pathology have been implicated or suspected in pulp stone formation,^2,4,26^ such as dental restorations,^5,25^ dental caries,^12^ dental trauma,^3^ history of orthodontic treatment,^12,33^ periodontitis,^9,17,34^ moderate to severe tooth wear,^31^ cardiovascular diseases,^2^ diabetes mellitus,^28^ and renal failure,^15^ among others. Additionally, age,^25^ gender,^10,12,25^ and genetic factors,^24^ may also influence the occurrence of pulp stones. Furthermore, the prevalence of pulp stones may vary across geographical regions and ethnic populations.^10^ A retrospective CBCT study by Zhang et al^36^ in Southwest China reported a pulp stone prevalence of 49% among individuals aged 16–45 years. Gao et al^6^ reported a prevalence of 26.2% in molars based on a CBCT analysis of individuals in Western China,while Xu et al^35^ documented a case of multiple pulp stones in an 11-year-old adolescent. Shandong Province, located in North China, is geographically distant from the western and southwestern regions mentioned above. To date, no data have been reported on the prevalence and distribution characteristics of pulp stones in North China.

In previous studies, when investigating the prevalence of pulp stones, local oral factors, systemic factors, and inherent characteristics were often analysed together.^5,8,12,25,36^ While this approach allowed for the examination of multiple variables, it could also lead to potential interference from other stimulating factors, which might obscure true occurrence and distribution characteristics of pulp stones. Therefore, this study established criteria to control for local and systemic factors and employed CBCT to observe the prevalence of pulp stones in a population from Shandong Province in North China. It aimed to investigate the associations of pulp stones with age, gender, and tooth position, and to analyse their occurrence and distribution patterns.

## METHODS AND MATERIALS

### Image Acquisition

Clinical data were retrospectively collected from patients who underwent CBCT examinations at the Department of Stomatology, Shandong Provincial Third Hospital, Shandong University, between October 2020 and January 2025, primarily for the assessment of impacted teeth. The study was approved by the ethics committee of the hospital (approval no.: KYLL-2025154). The inclusion and exclusion criteria were adapted and modified from previous studies.^6,23,24,32^ Inclusion criteria included: patients with 28 intact and developed permanent teeth (third molars excluded); CBCT scans of adequate diagnostic quality. Exclusion criteria included: patients with systemic disease (such as cardiovascular diseases, diabetes mellitus, and renal failure); CBCT scans with poor quality; dental, pulpal, or periapical disease; history of orthodontics treatment; history of dental treatment (including restorative and endodontic treatments); moderate to severe tooth wear (Scott’s scale No. 2–10)^31^; alveolar bone loss (> 2 mm from alveolar crest apical to cementoenamel junction)^9,34^; history of dental trauma (concussion, luxations, or fractures) associated with pain or discomfort^3,18^; third molars. A total of 335 patients who met the inclusion and exclusion criteria were enrolled, comprising 171 males and 164 females, aged between 20 and 40 years.

### Morphologic Analysis

CBCT images were acquired with patients in a standing position using a NEWTOM VGI (evo, Italy) scanner. The scanning parameters were set as follows: tube voltage 110 kVp, slice thickness 0.3 mm, and field of view 150 × 150 mm. Images were evaluated on a 21.5-inch LECOO M2211E (Lenovo, Beijing) monitor with a resolution of 1920 × 1080 pixels using the built-in NNT software. The pulp stones were identified as round or ovoid radiopacities located within the pulpal space (including both the crown and root).^5,24^

Data interpretation was conducted by two dentists with over 5 years of clinical experience. They assessed the presence of pulp stones from coronal, sagittal, and axial planes. Prior to the formal assessment, inter-observer reliability was tested using 50 images randomly selected from a separate, unrelated pool, yielding a Kappa value of 0.91. Subsequently, all images in the study were evaluated by both examiners. To assess intra-observer reliability, 50 images were randomly re-evaluated 1 month later, resulting in a Kappa value of 0.92. Any discrepancies arising during the formal evaluation were discussed between the two examiners until a consensus was reached. For each patient, the demographic information (including age and sex), the number of pulp stones, and their location were recorded.

### Statistical Analysis

Statistical analysis was performed using SPSS version 26.0. Categorical data were expressed as N (%). The chi-square test was employed to compare the prevalence of pulp stones across different genders, age groups, dental arches, and tooth positions. Generalised estimating equations (GEE) were applied to identify independent factors associated with pulp stone formation. Inter-observer reliability was assessed using the Kappa test. A *P* value < 0.05 was considered statistically significant.

## RESULTS

### Demographic Data

The study included 9,380 teeth (4,690 maxillary, 4,690 mandibular), distributed as 2,680 incisors, 1,340 canines, 2,680 premolars, and 2,680 molars. The overall mean age was 27.02 ± 5.89 years (males: 27.49 ± 5.87; females: 26.53 ± 5.89). Among the 335 patients, 95 (28.4%) presented with pulp stones (mean age 26.62 ± 6.22 years), including 56 females (25.93 ± 6.23) and 39 males (27.62 ± 6.04). A total of 585 teeth with pulp stones were identified, including 54 incisors, 70 canines, 36 premolars, and 425 molars.

### Distribution of Pulp Stones by Age and Gender Groups

A significantly higher prevalence was observed in females compared with males (34.1% vs 22.8%; *P* < 0.05), whereas no significant difference was found between the 20–30 and 31–40 year age groups (*P* > 0.05) (Table 1).

**Table 1:** Distribution of pulp stones by gender and age group

Variables	Patient with pulp stones N (%)	Patient without pulp stones N (%)	X^2^	*P*
Gender			5.298	0.021
Male	39 (22.8)	132 (77.2)		
Female	56 (34.1)	108 (65.9)		
Age (years)			0.017	0.895
20–30	67 (28.2)	171 (71.8)		
31–40	28 (28.9)	69 (71.1)		


### Number and Symmetry of Teeth with Pulp Stones

**Fig 1:**
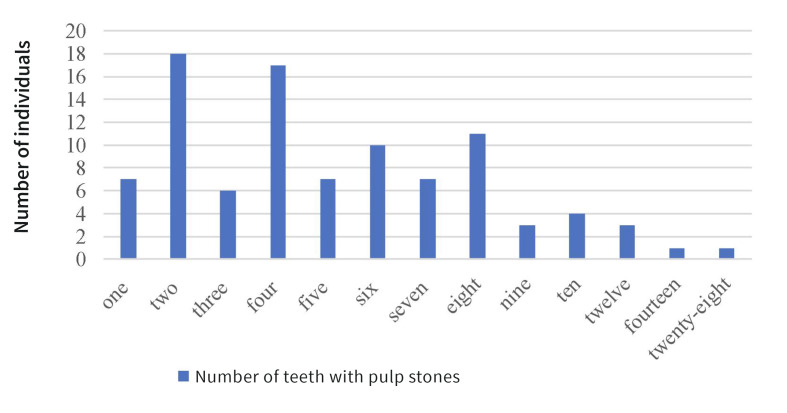
Distribution of individuals by number of teeth with pulp stones.

Among 95 patients with pulp stones, seven (7.4%) exhibited a single affected tooth (5 molars, 2 canines); 88 (92.6%) had two or more teeth involved. Notably, one 20- year- old female presented with stones in all 28 teeth. Of the 88 patients with multiple stones, 47 (53.4%) showed complete bilateral symmetry and only six (6.8%) were completely asymmetric (Fig 1).

### Distribution of Pulp Stones by Dental Arches and Tooth Types

**Fig 2a to d:**
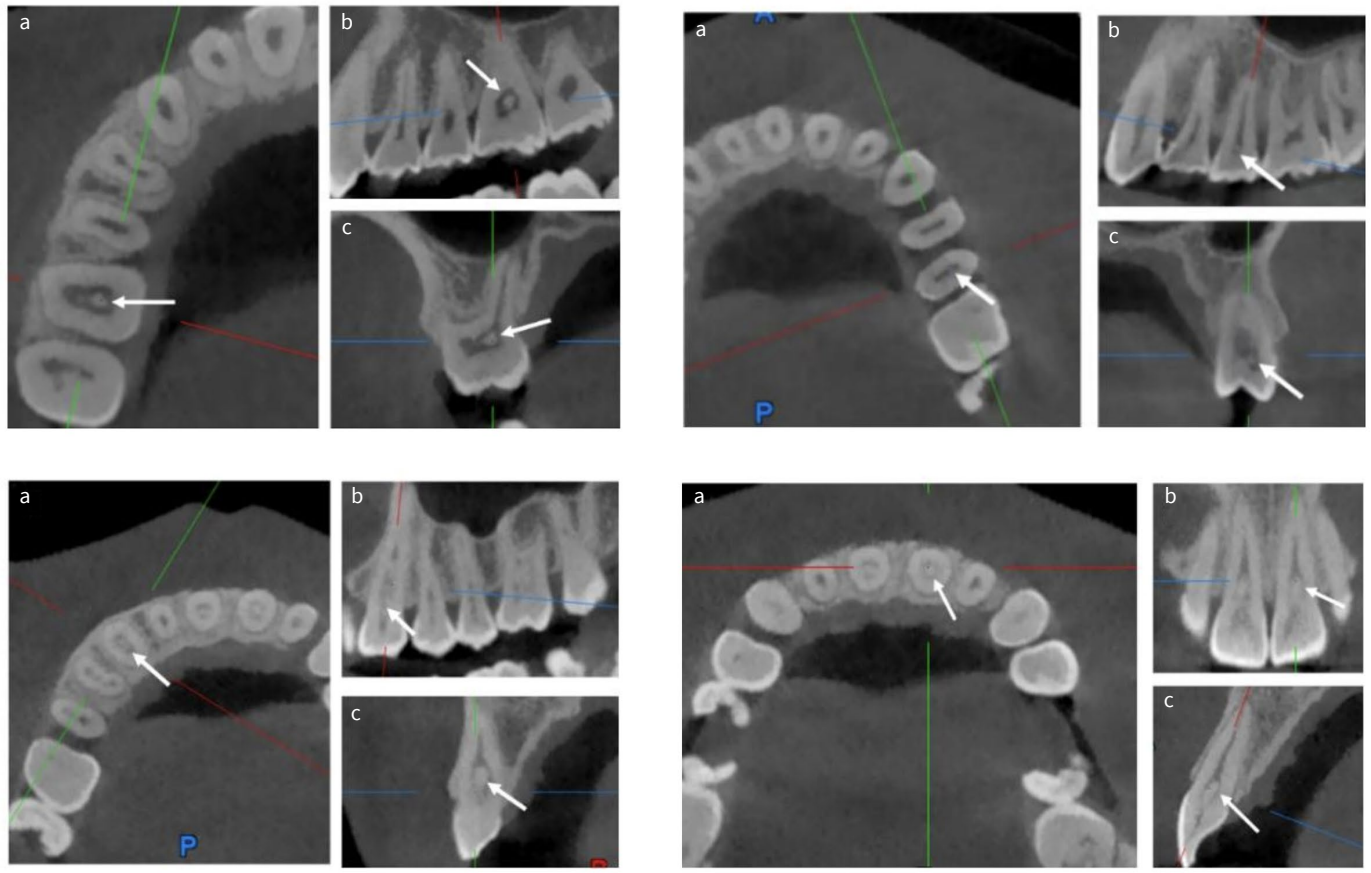

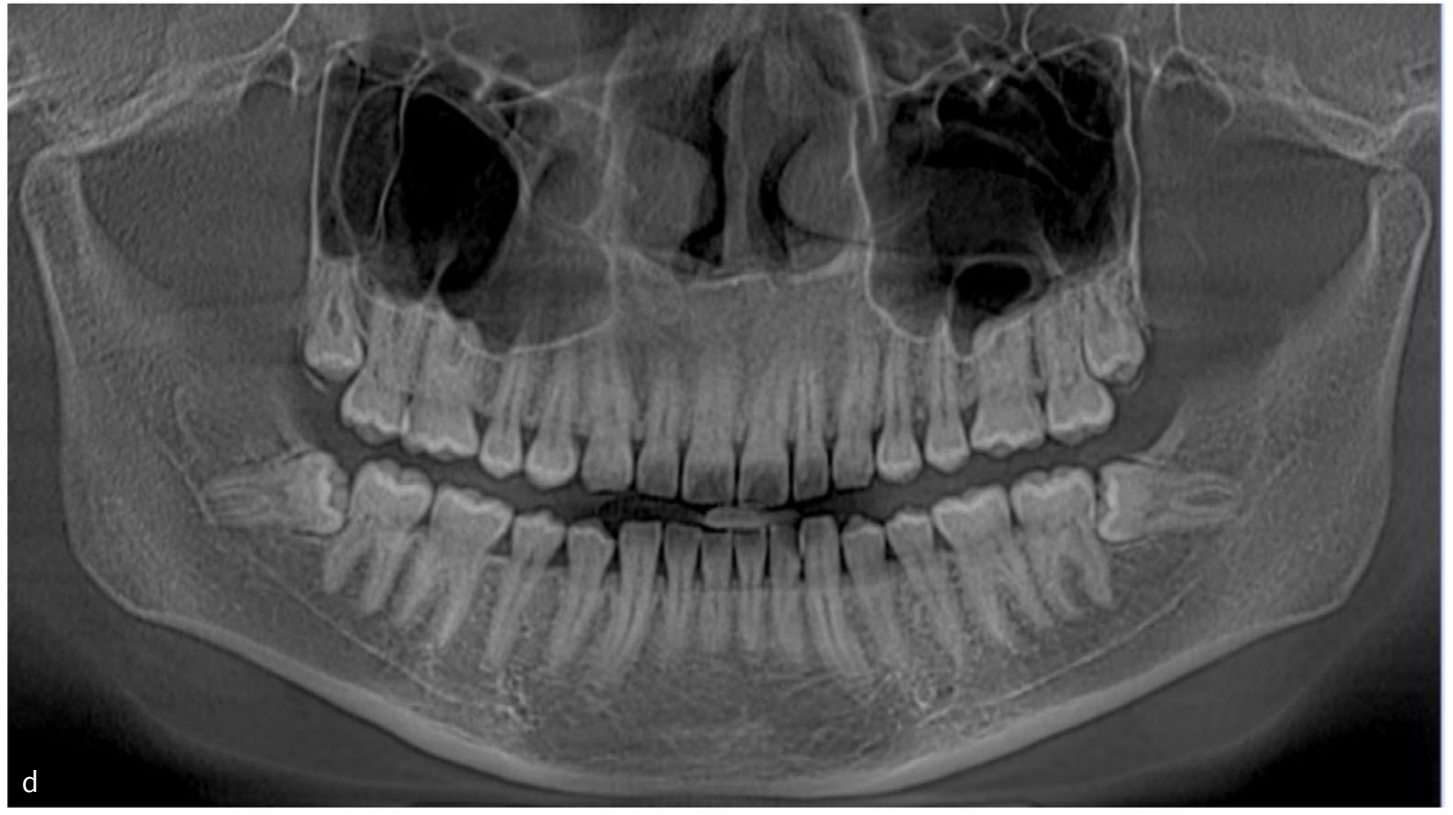
CBCT images of pulp stones in the right maxillary first molar, left maxillary second premolar, right maxillary canine, and left maxillary central incisor. Note: (a) axial view, (b) sagittal view, and (c) coronal view of the pulp stones. (d) Orthopantomogram (OPG) view of the CBCT images for the individual with pulp stones in all 28 teeth.

Pulp stones were present in 6.2% of teeth (585/9380). A significantly higher prevalence was observed in the maxillary arch than the mandibular arch (*P* < 0.05), while no significant difference was found between left and right sides (*P* > 0.05). Among tooth types, molars showed the highest prevalence (*P* < 0.001), exceeding that of premolars, canines, and incisors. Canines also exhibited a higher prevalence than incisors and premolars (*P* < 0.001), whereas no significant differences were found between incisors and premolars (*P* > 0.05) (Table 2). Figure 2 illustrates pulp stones in different tooth positions across coronal, sagittal, and axial CBCT views, including a panoramic reconstruction from a patient with pulp stones in all 28 teeth.

**Table 2:** Distribution of pulp stones by dental arch and tooth type

Variables	Teeth with pulp stones N (%)	Teeth without pulp stones N (%)	X^2^	*P*
Dental arch			4.027	0.045
Maxilla	316 (6.7)	4374 (93.3)		
Mandible	269 (5.7)	4421 (94.3)		
Side			2.773	0.096
Right	273 (5.8)	4417 (94.2)		
Left	312 (6.7)	4378 (93.3)		
Tooth type			618.040	0.000
Molars	425 (15.9)	2255 (84.1)		
Premolars	36 (1.3)	2644 (98.7)		
Canine	70 (5.2)	1270 (94.8)		
Incisors	54 (2.0)	2626 (98.0)		


Among molars, maxillary teeth showed a higher pulp stone prevalence than mandibular teeth, and first molars exceeded second molars (both *P* < 0.05), with no significant left- right difference. Maxillary first molars had the highest prevalence, significantly greater than mandibular first molars, maxillary second molars, and mandibular second molars (*P* < 0.008), while no significant differences were found among the latter three groups (Table 3).

**Table 3:** Distribution of pulp stones in the molar

Variables	Teeth with pulp stones N (%)	Teeth without pulp stones N (%)	X^2^	*P*
Dental arch			6.177	0.013
Maxillary molars	236 (17.6)	1104 (82.4)		
Mandibular molars	189 (14.1)	1151 (85.9)		
Side			0.003	0.958
Right molars	213 (15.9)	1127 (84.1)		
Left molars	212 (15.8)	1128 (84.2)		
Tooth type			11.815	0.001
First molars	245 (18.3)	1095 (81.7)		
Second molars	180 (13.4)	1160 (86.6)		
Tooth type			23.566	0.000
Maxillary first molars	145 (21.6)	525 (78.4)		
Mandibular first molars	98 (14.6)	572 (85.4)*		
Maxillary second molars	89 (13.3)	581 (86.7)^*^		
Mandibular second molars	91 (13.6)	579 (86.4)*		
*Compared with maxillary first molars, *P* < 0.001				

### GEE Analysis of Associated Factors with Pulp Stones

GEE analysis indicated significantly greater odds of pulp stone presence in the maxillary arch than the mandibular arch (odds ratio [OR] = 1.25, *P* = 0.021). Prevalence was significantly higher in females than in males (OR = 2.05, *P* = 0.004). Compared with incisors, the presence of pulp stones was significantly higher in the molars (OR = 19.28, *P* < 0.001) and canines (OR = 4.14, *P* = 0.002), while no significant difference was found between premolars and incisors (*P* > 0.05) (Table 4).

**Table 4:** GEE analysis of associated factors with pulp stones

Variables	aOR	95%CI of aOR	*P*
Dental arch			
Mandible	Ref.		
Maxilla	1.25	1.04–1.51	0.021
Gender			
Male	Ref.		
Female	2.05	1.26–3.34	0.004
Tooth type			
incisors	Ref.		
Canine	4.14	1.71–10.02	0.002
Premolars	1.20	0.45–3.21	0.712
Molars	19.28	7.98–46.56	< 0.001


## DISCUSSION

Pulp stones can form in healthy teeth, infected teeth, and unerupted teeth.20 Previous studies have predominantly focused on erupted teeth,^1,5,8,25,32^ with most analyses combining teeth subjected to local irritants (such as restorations or periodontitis) and healthy teeth. In contrast, Kalaji et al^13^ and Javaheri et al^11^ investigated pulp stones in unerupted teeth and reported a higher prevalence in unerupted canines and third molars, showing no significant association with gender but an increasing prevalence with age. The prevalence of pulp stones varies widely from 8% to 95% of the studied population,^16,32^ the prevalence in this study was 28.4%, which was lower than the 49% reported by Zhang et al.36 in a southwestern Chinese population aged 16–45 years. This difference may be attributed to our exclusion of patients with caries, restorations, or other local oral factors.^27^ The prevalence at the tooth-level (6.2%) was close to their reported 7.4%.^14^ As for gender differences, females exhibited significantly higher odds of pulp stones than males, a result comparable to the odds ratio of 1.9 reported by Zhang et al.^36^ Jannati et al^10^ also supported a higher prevalence in females compared with males, a difference that may be associated with oestrogen levels.^1^

Consistent with Hsieh et al,^8^ who found no significant age-related association in individuals over 20 years in northern Taiwan, our study also showed no significant differences across age groups. While previous research suggested that ageing itself may not be a primary cause – instead attributing formation to cumulative pulp stimuli (eg, caries) and age-related systemic diseases (eg, diabetes) – other work proposed that ageing promotes calcification through degenerative pulpal changes,^22^ such as reduced cellularity, progressive secondary dentin deposition, and possible lipid accumulation.^21^ The age range examined here may represent a plateau phase in pulp calcification, during which metabolic activity in mature pulp cells remained relatively stable. Further studies across wider age ranges are needed to clarify the role of age in pulp stone formation.

Notably, 92.6% of patients presented with two or more pulp stones, in agreement with the observation by Goga et al^7^ that pulp stones can affect a single tooth or the entire dentition. Here, nine patients had pulp stones in more than 10 teeth, including one 20-year-old female with stones in all 28 teeth, where large calcified masses obstructed the pulp chambers and root canal orifices in multiple teeth. This aligned with the finding by Sener et al^24^ that 1% of patients exhibited pulp stones in over 10 teeth. Evidence also suggested that multiple pulp stones may be associated with familial predisposition.^19^ Additionally, 53.4% of affected patients showed completely bilateral symmetric distribution of teeth with pulp stones, indicating a tendency for symmetry within individuals.

When considering dental arches, the present study found a higher prevalence of pulp stones in the maxilla than in the mandible, consistent with previous studies,^27,36^ but in contrast to the report by Kannan et al^14^ who found no significant difference between arches, a discrepancy highlighting the need for larger- scale studies. In line with Kannan et al,^14^ no significant left–right difference was observed.

In terms of tooth types, molars showed the highest prevalence of pulp stones due to their anatomical and physiological features, such as large size, abundant pulp tissue and blood supply, strong reparative capacity, and substantial masticatory load.^5,8^ First molars were notably highly affected, likely because of their early eruption and longer exposure to occlusal stimuli.^10^ Further analysis revealed a higher prevalence of pulp stones in maxillary first molars than in mandibular first molars. Gao et al^6^ suggested this may be related to the typically three-rooted morphology of maxillary molars, which could provide a richer pulpal blood supply compared with two-rooted mandibular molars – a hypothesis that warrants further investigation.

Furthermore, canines were more frequently affected than incisors and premolars. Zhang et al^36^ reported a higher prevalence of pulp stones in anterior teeth (2.8%) compared with premolars (1.2%), with the majority of stones located in the middle and upper thirds of canine root canals. Javaheri et al^11^ found a higher prevalence of pulp stones in unerupted canines, although the underlying aetiology remains unclear. Contrary to our results, Sezgin et al^25^ observed that premolars were more frequently affected than canines and incisors, with the discrepancy potentially related to differences in patient demographics between the study populations.

This study implemented several measures to minimise potential biases. Detection bias was addressed with a pilot study, intra- and inter-examiner reliability assessments, and consensus discussions to resolve discrepancies. Strict inclusion/exclusion criteria controlled confounding factors. Information bias was minimised by using only complete, high-quality records and cross-verifying all data. These approaches enhanced the study’s precision and reliability.

Several limitations of this study should be acknowledged, including its single-centre retrospective design, restricted age range and geographical coverage, and unassessed factors like genetic predispositions. While the strict criteria controlled for confounding, they may also limit generalisability, underscoring the need for future multi-centre or longitudinal studies with larger, diverse samples.

## CONCLUSION

In summary, pulp stones were more commonly found in females, maxillary teeth, and molars among young and middle-aged populations in North China, with the highest frequency observed in maxillary first molars. No significant association was found between pulp stone occurrence and either age or the left–right side of the dental arch. Additionally, multiple occurrences and symmetric distribution patterns of pulp stones were demonstrated within individuals.

### Data Availability Statement

The original contributions presented in the study are included in the article/Supplementary Material; further inquiries can be directed to the corresponding author.

#### Ethics statement

The study has been approved by the ethics committee of Shandong Provincial Third Hospital affiliated to Shandong University (Approval No.: KYLL-2025154). The studies were conducted in accordance with the local legislation and institutional requirements.

#### Funding

The author(s) declare that no financial support was received for the research and/or publication of this article.

#### Conflict of interest

The authors declare that the research was conducted in the absence of any commercial or financial relationships that could be construed as a potential conflict of interest.
